# Design and Operation of the Lombardy Parkinson's Disease Network

**DOI:** 10.3389/fneur.2020.00573

**Published:** 2020-06-26

**Authors:** Alberto Albanese, Alessio Di Fonzo, Vincenza Fetoni, Angelo Franzini, Michele Gennuso, Graziella Molini, Claudio Pacchetti, Alberto Priori, Giulio Riboldazzi, Maria Antonietta Volonté, Daniela Calandrella

**Affiliations:** ^1^Department of Neurology, IRCCS Humanitas Research Hospital, Rozzano, Italy; ^2^Neurology Unit, Foundation IRCCS Ca' Granda Ospedale Maggiore Policlinico, Milan, Italy; ^3^Neuroscience Section, Department of Pathophysiology and Transplantation, Dino Ferrari Center, University of Milan, Milan, Italy; ^4^Neurology Department, ASST Fatebenefratelli Sacco, Milan, Italy; ^5^Department of Neurosurgery, IRCCS Istituto Neurologico Carlo Besta, Milan, Italy; ^6^ASST Ospedale Maggiore di Crema, Crema, Italy; ^7^ASST Melegnano e Martesana, UO di Neurologia, Vizzolo Predabissi, Italy; ^8^Parkinson and Movement Disorder Unit, IRCCS Neurological Institute “C. Mondino” Foundation, Pavia, Italy; ^9^Movement Disorders Clinic, III Clinica Neurologica, Università degli Studi di Milano, Dipartimento di Scienze della Salute, San Paolo University Hospital, Milan, Italy; ^10^“Aldo Ravelli” Research Center for Neurotechnology and Experimental Brain Therapeutics, University of Milan, Milan, Italy; ^11^ASST Santi Paolo e Carlo, Milan, Italy; ^12^Parkinson's Disease and Movement Disorders Center, ASST Sette Laghi, Varese, Italy; ^13^Department of Neurology, IRCCS San Raffaele Scientific Institute, Milan, Italy

**Keywords:** Parkinson disease, health maintenance organizations, disease management, managed care programs, consensus

## Abstract

**Background:** Parkinson's disease (PD) is one of the most common chronic neurological conditions leading to disability and social burden. According to the 2016 Italian National Plan on Chronic Diseases, regional health authorities are implementing dedicated networks to manage neurological diseases, including PD.

**Methods:** A panel of experts representing health-care providers in Lombardy reached consensus on the organization of a patient-centered regional PD healthcare network.

**Results:** The panel proposed a structure and organization implementing a hub-and-spoke PD network model. Three levels of neurological services were identified: General Neurologist, PD Clinic, PD Center. This model was applied to health service providers currently accredited in Lombardy, yielding 12 candidate PD Centers, each serving an area of ~1,000–2,000 km^2^, and not less than 27 PD Clinics. The panel agreed on uniform diagnostic and staging criteria for PD, and on a minimum common clinical data set, on PD patient management by the network at initial and follow-up assessments, on the cadence of follow-up visits, on patient referrals, and on outcome measures for the assessment of network activities.

**Conclusions:** The implementation of disease-centered networks for chronic neurological diseases provides an innovative opportunity to improve patient management, facilitate research and education.

## Introduction

Aiming to improve multidisciplinary management and reduce inhomogeneity of interventions, the 2016 Italian National Plan on Chronic Diseases stated that each regional health system must establish health networks dedicated to the management of chronic diseases (1). The implementation of this plan started last year and was recently delayed by the outbreak of Covid-19 epidemic. Parkinson's disease (PD) is one of the most common neurological conditions and constitutes a model of treatable chronic neurological disease. It is characterized by a variety of neurological and non-neurological features and progresses variably towards a stage of social burden and disability (2). Management of PD requires specific medical expertise and dedicated resources.

In Italy regional health authorities are the payers for the National Health System (NHS); they finance health-care providers (HCPs) through yearly budget plans. A disease-centered regional PD network is expected to reduce inequalities in treatment and to harmonize the use of regional resources. A panel of neurologists with expertise on PD reviewed the available evidence and developed a consensus on the set-up and the general organization of a regional PD network. The primary objective was to improve PD patient care while optimizing resources, a secondary objective was to facilitate patients' participation in research programs through the network.

## Materials and Methods

As a preparatory work, the Directorate General for Health collected data related to medical certificates, prescriptions, and hospital admissions from 2012 through 2017. The following data were retrieved from the electronic dataset: prevalence and incidence of PD cases, acute admission of PD patients to Lombard hospitals, consumption of antiparkinsonian medications, and outpatient consultations for PD patients.

A panel of neurologists representing Lombard HCPs with expertise on PD was convened. The represented HCPs encompassed three private and seven public institutions, four universities, five research hospital, and three general hospitals. The panel was composed by neurologists responsible for PD care in each of the participating HCPs, who were asked to design a general model of the regional PD network. The methodology for nominal group consensus process was implemented, involving the following principles: all members contribute to the discussion, can state each issue in their own words, have the opportunity and time to express their opinion about each issue, and agree to take responsibility for the implementation of a decision.

The panel reviewed the established PD networks in Italy and Europe and took into account the Lombard guidelines on healthcare networks (3) that apply to all chronic diseases. The list of accredited Lombard HCPs was downloaded from the Lombardy HCP repository. Hospitals and ambulatory care clinics were included, whereas rehabilitation centers and assisted living residences were excluded. For each HCP with neurological facilities expertise on PD was assessed and ranked. The network structure was outlined and the PD patient's journey through the network was assessed before drafting a final consensus.

A first draft of the manuscript was prepared based on the results of data analysis, discussion, and comments from panel members. To reach the final consensus, the last draft and the preliminary conclusions were critically discussed with representatives from PD patient associations.

## Results

The Lombardy regional health service is managed through eight territorial branches, spanning from the northern mountainous regions, through urbanized areas in the middle region, to plains in the south. In 2016 there were 36,217 PD patients and 10,036,258 residents, yielding an annual prevalence of 277 cases per 100,000, in the high range of epidemiological findings in Europe (4). The regional dataset showed an 11% increase in PD prevalence from 2012 to 2017. The number of patients with lower burden of concomitant chronic diseases increased by 55.5% and those with a higher burden by 16%. Acute admissions to hospitals did not vary over years. In general, about a half of outpatient consultations were neurological, half with other specialists (Table 1).

**Table 1:** Data on PD patients in Lombardy (years 2012–2017).

**Variable measured**	**2012**	**2013**	**2014**	**2015**	**2016**	**2017**
Prevalence of PD patients	33,109	33,844	34,458	34,934	36,217	36,637
Prevalence of PD patients stratified by regional burden scale (3)						
• High burden	10,251	10,593	10,895	11,116	11,426	11,882
• Intermediate burden	7,991	7,909	7,973	7,917	8,073	7,951
• Low burden	690	775	851	904	1,036	1,073
Incidence of PD cases (per 100,000 inhabitants)	12.7887	12.6222	13.4328	13.3789	13.1253	11.3656
Acute admissions to hospitals (number per 1,000 resident population)	0.21	0.20	0.20	0.21	0.18	0.19
Consumption of antiparkinsonian medications (defined daily dose)	1,256.9531	1,244.8152	1,299.4366	1,311.0817	1,337.9097	1,101.3466
Outpatient consultations related to PD (number per 1000 resident population)	44.07	45.49	45.16	44.99	43.43	42.44
Outpatient consultations related to PD (total number)						
• Neurology	37,564	37,570	38,250	37,482	37,874	37,596
• Ophthalmology	7,888	7,804	7,714	7,510	7,544	7,462
• Orthopedics	7,010	7,016	7,350	7,318	7,030	7,328
• Physical medicine	4,140	4,558	4,616	4,796	4,998	5,222
• Endocrinology	4,250	4,732	4,790	4,730	4,690	4,786
• Cardiology	3,898	3,988	3,908	3,752	3,612	3,751
• Otolaryngology	3,043	3,228	3,120	3,210	3,269	3,213
• Urology	2,930	2,793	2,935	3,008	3,057	3,248

*PD, Parkinson's disease*.

This analysis supported the need to design a patient-centered regional network to serve as a basis for building a multidisciplinary PD management in the Lombardy region.

### Neurological Facilities for PD

The management of PD patients in the early and advanced stages involves a variety of health settings, according to disease progression and to changes in patients' needs. The panel recognized that three main neurological settings are involved in the management of PD patients: General Neurologists, Parkinson Clinics and Parkinson Centers.

**General Neurologists** see a variety of neurological patients, whom they occasionally refer to specialized neurological services, particularly if there is need to manage complications or medical emergencies. General Neurologists see also PD patients, usually until the advanced disease stage. In addition, outpatient services with dedicated expertise on PD, called **PD Clinics**, look after PD outpatients and deploy specific skills for the management of the advanced stages. Finally, more articulated settings, called **PD Center**s, implement complex diagnostic and treatment protocols on PD patients. Non-neurological consultations involve the patient's general practitioners and specialists outside neurology: unless strictly connected with neurological centers, they may lack expertise on the specific needs of PD patients and on the possible interactions of antiparkinsonian medications.

The panel provided a definition for **PD Clinics**. They are outpatient neurology services of a hospital or an ambulatory neurology care clinic devoid of inpatient facilities, whose dedication to PD is recognized at administrative level (by the regional or the HCP administration). PD Clinics include at least one neurologist with post-residency training in movement disorders. Here PD patients receive assessment and personalized prescription of specific PD treatments. PD Clinics provide care across the full spectrum of patients' needs, including motor, non-motor and cognitive assessment.

The panel implemented the profile of a higher level network centers outlined by Lombard regional guidelines (3) and took into consideration also the definition of PD excellence centers provided by non-governmental organizations. **PD Centers** are defined as hospital HCPs which: (1) have a neurological ward; (2) deliver care according to a coordinated team model and include two or more neurologists with post-residency training in PD and movement disorders; (3) have taken in charge at least 700 unique patients with parkinsonism in the last 12 months; (4) regularly perform interventional treatments for advanced PD; (5) implement national and international research programs related to PD; (6) provide educational programs related to PD; (7) implement multi-disciplinary and multi-professional PD care; (8) receive referrals of patients with complex or rare PD variants.

**Figure 1:**
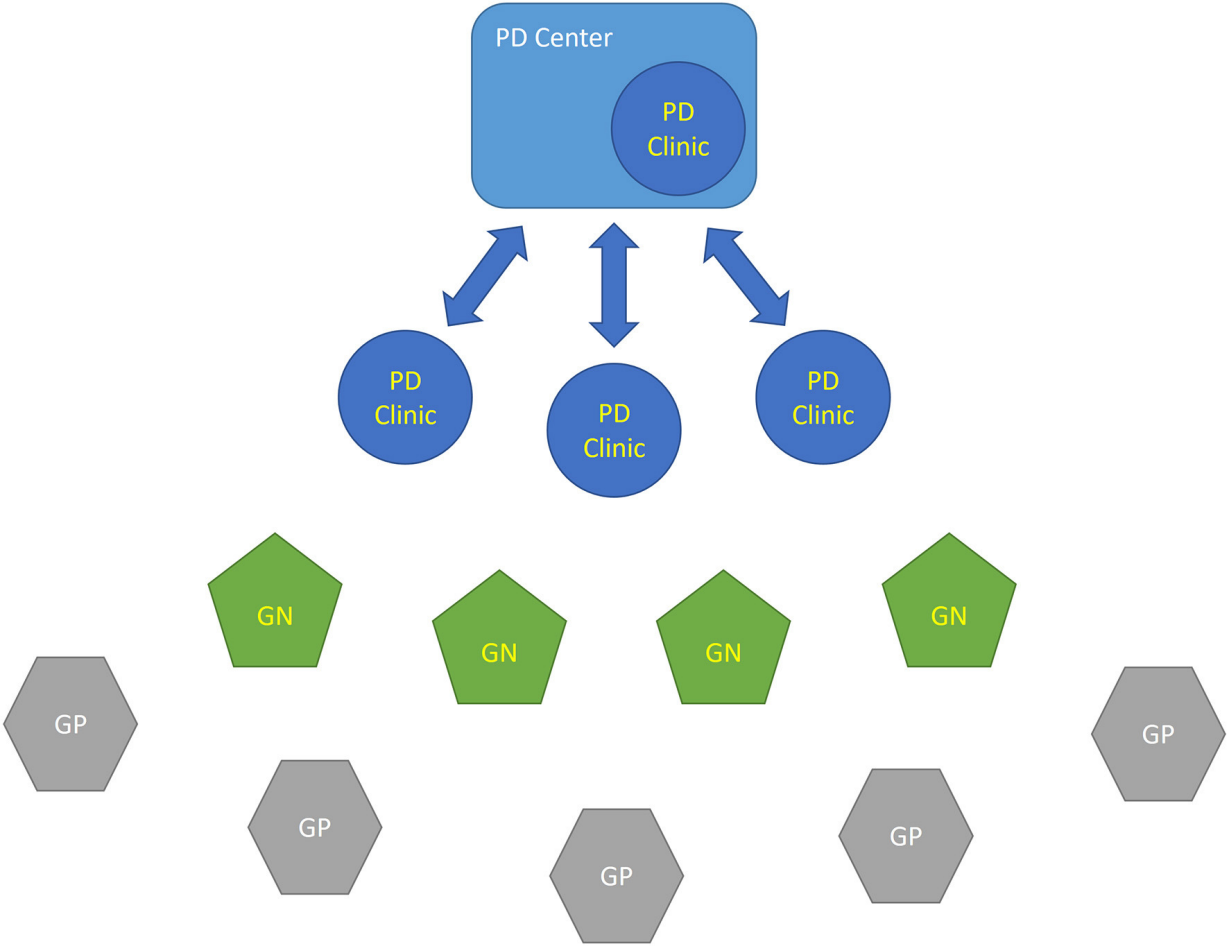
Schematic relationship between different HCP centers within the PD network. The patient's journey is indicated by lines. A PD Center is a high level excellence center for PD with multidisciplinary inpatient facilities and an outpatient PD Clinic. PD Clinics, General Neurologists (GNs) and General Practitioners (GPs) interface with a PD Center in order to address patients' needs or contribute to research programs. GN, General Neurologist; GP, General Practitioner; PD, Parkinson's disease.

**Figure 2:**
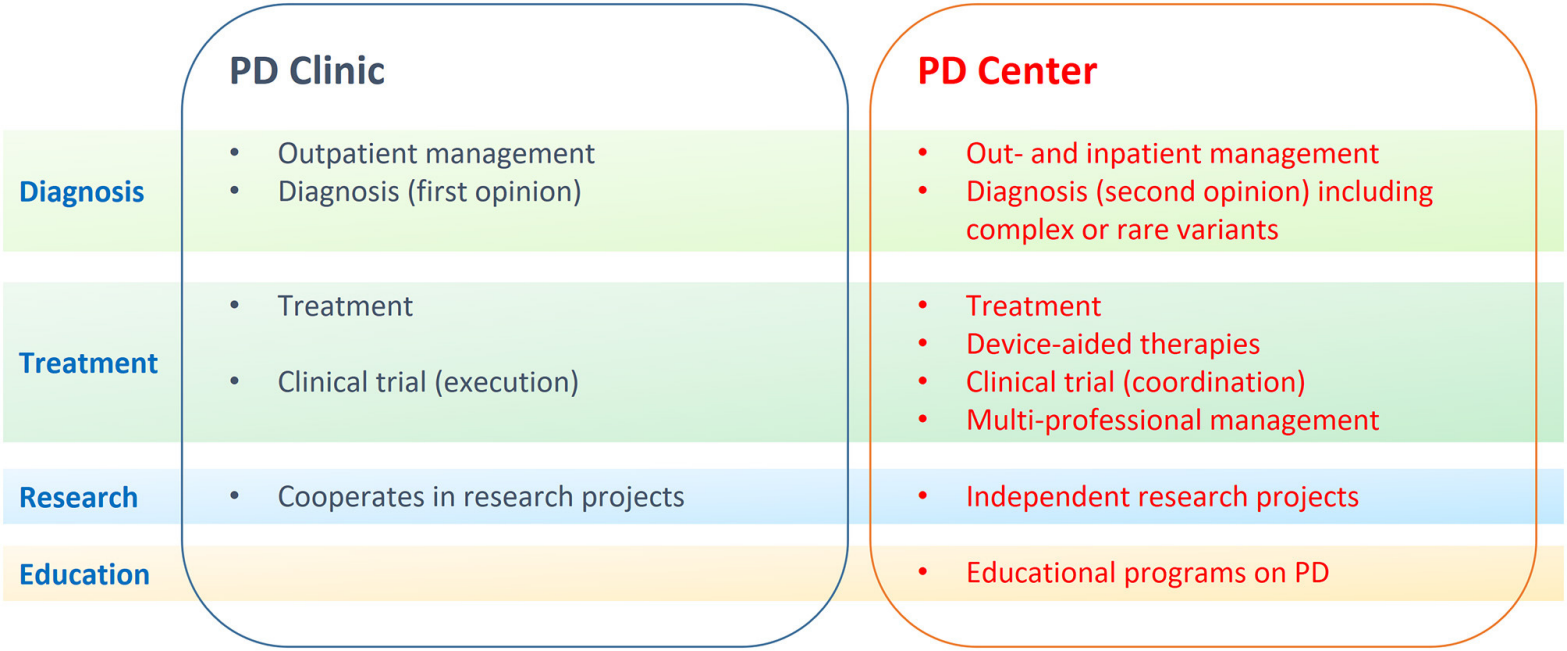
The different competences between Parkinson Disease (PD) Centers and Clinics are outlined. PD Centers and PD Clinics interact and cooperate on patient-centered issues at different levels: diagnosis (green), treatment (purple), research (blue), and education (yellow).

The panel agreed that a regional PD network is composed by two levels of specific expertise: PD Centers and PD Clinics that interact with General Neurologists, General Practitioners and other health professionals to deliver high standards of care to PD patients (Figures 1, 2).

Review of HCPs accredited by the NHS in the Lombardy region reported 322 ambulatory care clinics (142 public, 180 private), which deliver neurological consultations, but have no inpatient facilities. There are 232 hospitals (106 public, 126 private): 183 are general hospitals (including 12 university hospitals), 19 are research hospitals endorsed by the Ministry of Health (5 public, 14 private). Neurological services are present in 94 general/university hospitals and in 10 research hospitals (2 public and 8 private; Table 2). Interventional treatments for advanced PD are currently offered by 11 general/university hospitals and by six research hospitals; deep brain stimulation (DBS) is offered by 11 hospitals, enteral levodopa infusion by 14 hospitals.

**Table 2:** Accredited HCPs listed in the official repository of the Lombardy region (see methods).

	**HCPs with neurology service**		**Location**	
	**Public**	**Private**	**Milan metropolitan area**	**Outside Milan**
Outpatient clinic	142	180	122	200
General hospital/university hospital	42 (6)	52 (1)	24 (3)	70 (4)
Research hospital (IRCCS)^*^	2 (2)	8 (3)	8 (4)	2 (1)

* *The number of potential PD Centers is reported in brackets. Istituto di Ricovero e Cura a Carattere Scientifico (IRCCS)*.

**Figure 3:**
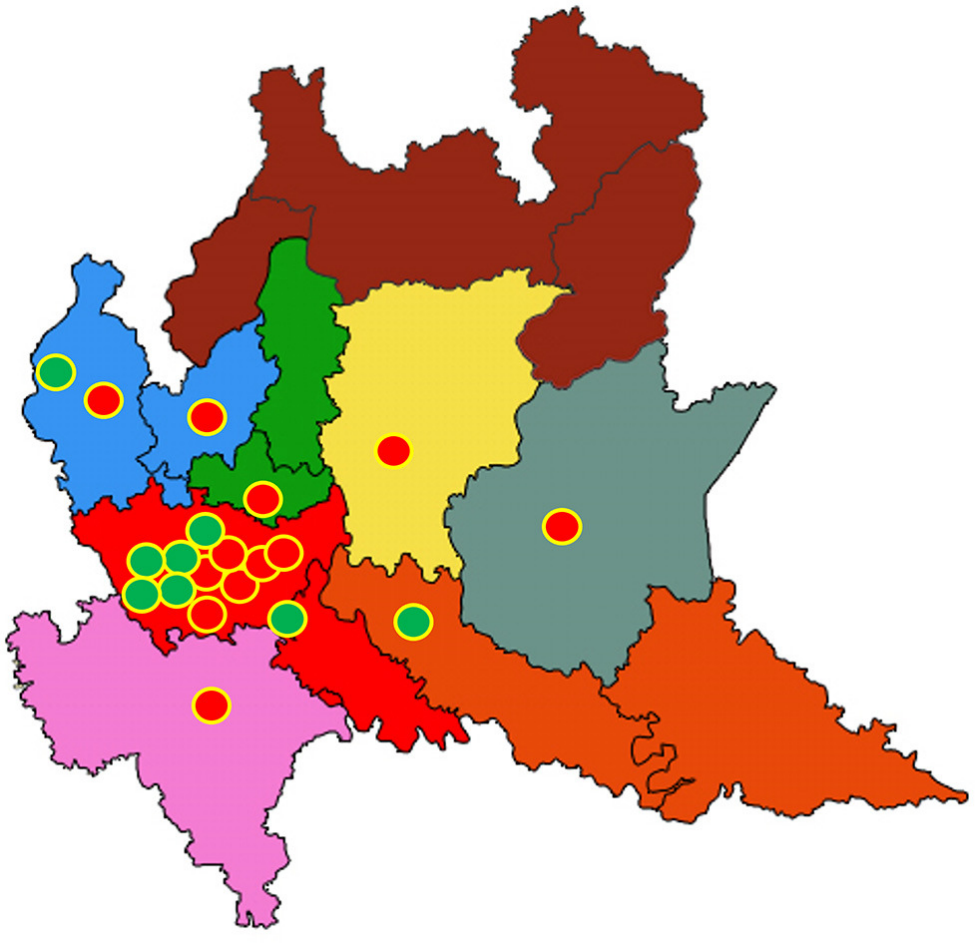
Geographic distribution of potential Parkinson Disease (PD) Centers in the Lombardy region. Twelve health care providers (HCPs) fulfilling strict criteria for PD Centers are represented by red circles; eight additional HCPs fulfilling more lenient criteria are shown by green circles. The eight territorial branches of the Lombardy region Health Directorate are shown by different colors. Provincial districts are shown by lines. See text for further details.

It was reckoned that ~12 centers in the Lombardy region meet all criteria for a PD Center, whereas approximately eight additional centers meet all but one criterion (Figure 3). The latter group includes HCPs that only implement one type of advanced treatment (usually enteral levodopa infusion) or do not perform clinical trials. Considering that the Lombardy region covers a surface of 23,844 km^2^, each PD Center would serve a geographic area of 1,000–2,000 km^2^ and would interconnect with up to 27 network PD Clinics.

### Disease Progression

Progression of PD is marked by an increase in severity of motor symptoms, the emergence of levodopa-induced motor complications and the occurrence of dopaminergic resistant non-motor phenomena. This motor progression is nonlinear, with a variable speed of decline in motor and non-motor functions (5). The Hoehn and Yahr staging system, which combines functional disability with objective impairment, is commonly used to measure disease progression (6). When a patient reaches stage 3, risk of dementia increases, survival expectation decreases, and the total Unified PD rating scale (UPDRS) scores increase despite drug adjustment (6). Late stage PD is defined as stages 4 and 5 on the Hoehn and Yahr scale, which correspond to a stage with a progressive loss of physical independence that is irreversible in most patients (7).

The Lombardy regional guidelines ranked the burden of chronic diseases according to three levels, depending to the number of concomitant chronic diseases, their severity and the patient's dependence (3). The panel agreed that there is no reliable tally between the classification of burden proposed by the Lombardy region and PD staging used in neurological practice. For the purpose of clinical management, it was agreed to identify three PD stages [early, advanced and late (8)]. The early stage is characterized by mild symptoms and minimal or no functional impairment; the advanced stage denotes patients with motor complications. The late PD stage, instead, defines patients who are highly dependent on caregivers for ADL, owing to treatment-resistant motor or non-motor symptoms. Late-stage PD patients have a higher burden of chronic disease, are less manageable by the PD network, and often reside at residential and home care facilities.

A simplified approach suggests that early PD patients are prevalently seen by General Neurologists or at PD Clinics, advanced PD patients may need some consultation at PD Centers, and patients in the late stage will prevalently be seen by General Practitioners and referred to General Neurologists. PD Clinics and PD Centers may provide specific consultations to late stage PD patients when deemed relevant. A dynamic interaction between PD Clinics and PD Centers is considered a strength of the regional network at all disease stages. It is reckoned that early PD patients, if seen by a General Practitioner or by a General Neurologist, will be referred at least once to a PD Clinic to facilitate their enrollment in dedicated clinical trials. PD Clinics and PD Centers are competent to prescribe genetic and other specialized testing, when appropriate, to diagnose atypical cases.

### PD Network

The network is a patient-centered model of care, connecting high specialty centers (PD Centers) to less specialized centers scattered throughout the network territory. A first aim is to treat PD patients consistently throughout the regional territory, to offer homogeneity of treatment and access to more specialized care whenever needed. A second aim is to avoid unnecessary fragmentation, repetition or delays in diagnosis and treatment of PD patients. A third aim of the network is to facilitate scientific programs by developing active interconnection among centers dedicated to PD. The network structure depicts a patient's journey guided by clinical decisions that combine scientific and methodological rigor, quality of care, fairness of performance, diagnostic, and therapeutic appropriateness.

### Network Operations

The network takes charge of patients with PD symptoms based on referral from a General Practitioner, a General Neurologist or the patient himself.

### Entry Visit

The main purpose of the first visit at a PD Clinic is to define diagnosis. The PD network implements current diagnostic criteria for PD set by the International Parkinson Disease and Movement Disorders Society (9) to establish a patient's diagnosis upon entry. According to these criteria, patients may receive one of the following clinical diagnoses: Clinically established PD, Clinically probable PD, Parkinsonism (likely not PD), Non-parkinsonism.

A personalized set of diagnostic tests is performed based on the patient's clinical presentation. Diagnostic tests include morphologic neuroimaging (brain MRI or CT), functional neuroimaging (DAT scan, FDG-PET, etc.), genetic assessment (NGS panel or individual gene testing), neuropsychological assessment, autonomic assessment, vascular, or systemic workup. The neurologist in charge of the entry visit identifies the appropriate diagnostic tests. In case of uncertainty, the patient is referred to a PD Center within the network.

Patients who receive a diagnosis of clinically established or probable PD are assessed using the following tools: MDS-UPDRS scale (10), the non-motor symptoms scale (11), and the modified Hoehn and Yahr scale (6). The patients are classified as having early-, advanced-, or late-stage PD (8).

Patients who receive a diagnosis of Parkinsonism (likely not PD) are further assessed for alternative diagnoses fitting current diagnostic criteria for parkinsonian syndromes other than PD, such as multiple system atrophy (12), progressive supranuclear palsy (13), corticobasal degeneration (14), etc. Patients who do not fit with any diagnosis alternative to PD are subject to a full diagnostic reassessment upon follow-up.

Patients who are denied a diagnosis of parkinsonism are evaluated again for diagnosis upon request by their general practitioner. Table 3 summarizes the assessments performed at each network visit depending on the clinical diagnosis.

**Table 3:** Diagnosis and staging of PD patients as assessed by network centers.

**Assessment**	**Clinically established PD**	**Clinically probable PD**	**Parkinsonism (likely not PD)**	**Non parkinsonian**
MDS-UPDRS	✓	✓		
NMSS	✓	✓		
Hoehn-Yahr staging	✓	✓	✓	
Early/advanced/late staging	✓	✓	✓	
Other disease-specific rating scales			✓	
Treatment information	✓	✓	✓	
Genetic panel	✓		✓	
Imaging	✓	✓	✓	✓

*MDS-UPDRS, Movement Disorders Society Unified Parkinson's disease rating scale; NMSS, Non-motor symptoms scale*.

### Follow-up Visits

Patients with a diagnosis of clinically established PD are followed-up every 6 to 12 months, depending on clinical stage and comorbidity. Patients with a diagnosis of clinically possible PD are followed-up every 6 months and reassessed for diagnostic refinement. Patients with a diagnosis of non-PD parkinsonism, who do not have an alternative diagnosis, are reassessed every 3 to 6 months, depending on the clinical stage and comorbidities. Patients with uncertain response to chronic dopaminergic treatment may receive an acute levodopa challenge test (15).

At each follow-up visit, all patients are subject to a quick diagnostic reassessment that is expected to be confirmatory in most instances; but may occasionally lead to reconsider the diagnosis. Additional diagnostic testing may be prescribed at this stage, particularly to patients with a diagnosis of clinically probable PD, in order to refine the diagnosis or reconsider the prescribed treatment.

### Patient Referrals

A valuable network facilitates referrals between participating centers and with outside practices. Referrals within the network may have different motivations, such as: (1) seeking expert advice on a diagnostic or treatment issue; (2) requesting device-aided treatments unavailable at the referring center; (3) enrolling patients in clinical trials. Referrals from a PD Clinic are expected to be addressed to a neighboring PD Center, although there should be freedom to contact any network center.

All network centers should meet at least once a year, in order to align standards of care and share information on experimental trials and new procedures. Each PD Center should organize at least another yearly meeting with the neighboring PD Clinics to review referrals and patient outcomes. Significant changes in medical staff may require realignment of practices or update of operational standards during dedicated network meetings.

Outside referrals are mainly related to comorbid conditions that are best treated by a non-neurological specialist practice (Table 4). Patients with a high comorbidity burden are likely to be seen by multiple centers, including those within the PD network. Most commonly, PD Clinics and PD Centers refer patients outside the network to obtain a multidisciplinary consultation for non-motor or systemic symptoms. Assessment of non-motor symptoms with the non-motor symptoms scale (11) is a prerequisite to referring PD patients to non-neurological centers. The PD network maintains a database of non-neurological specialist centers that have expertise on PD.

**Table 4:** Main non-motor symptoms leading to referrals of PD patients to specialized practices outside the PD network.

**Non-neurological specialist practice**	**Main non-motor symptoms**
Ophthalmologist	Blurred vision, diplopia
Orthopedist	Shoulder pain, back pain
Physical therapy specialists	Freezing of gait, loss of postural control, falls
Endocrinologist	Diabetes, thyroid dysfunction
Cardiologist	Cardiac dysrhythmias, postural hypotension, blood pressure variability
Otolaryngologist	Dysphagia
Urologist, gynecologist, andrologist	Urgency, incontinence, sexual dysfunction
Neuropsychologist	Cognitive dysfunction, dementia
Psychiatrist	Anxiety, depression
Gastroenterologist	Constipation, digestive problems
Nutritionist	Overweight or underweight

*Non-neurological consultations are listed in decreased order of frequency based on data published by the Lombardy region (see Table 1)*.

### Device-Aided Therapies

Device-aided (also called interventional or advanced) therapies allow to manage PD patients with a treatment potential, whose motor symptoms cannot be controlled adequately by oral medications. The main reason for addressing a patient to device-aided therapies is the occurrence of PD-related fluctuations and dyskinesias that change the patient's conditions during the day, often abruptly or unpredictably.

Having reached the advanced stage of PD does not necessarily mean that a patient is fit for device-aided therapies. Suitable patients are rather a sub-group of patients with advanced PD. A set of clinical criteria for addressing patients to device-aided treatments has been recently defined (16). The panel reached consensus on a simplified list of clinical features favoring or disfavoring device-aided therapies for PD (Table 5). These treatments currently encompass DBS and enteral levodopa. New device-aided treatments are under development, including subcutaneous levodopa delivery, intrathecal infusion of anti-sense oligonucleotides, cell-based approaches, and viral gene delivery (17).

**Table 5:** Main indications and contraindications to device-aided therapies.

**Favor device-aided therapy**	**Disfavor device-aided therapy**
• Excellent and sustained levodopa response	• Dysphagia
• Levodopa resistant tremor	• Freezing of gait (OFF-related)
• Troublesome dyskinesia	• Dysarthria
• Pain	• Psychosis
• Intact cognitive function	• Dementia
• Night-time sleep disturbances	• Apathy
• Impulse control disorder	• Hallucinations
• Depression	• Postural impairment and gait disturbance
• Anxiety	• Older age (>70)
• Limitation with ADLs	• Insufficient compliance
• Younger age (<70)	• Lack of caregivers
	• Living in a nursing home

General Neurologists or PD Clinics select patients for device-aided treatments and refer them to a PD Center, where the indication is reviewed and the most appropriate treatment is implemented according to current guidelines. At the end of the procedure, the patient is readdressed to the referring center for follow-up visits. As a rule, changes in stimulation settings are performed by PD Clinics and PD Centers; General Neurologists can test PD patients with stimulation on or off and adjust the infusion rate of enteral or subcutaneous antiparkinsonian medications. Based on specific protocols, particularly for research purposes, some follow-up visits may be performed at the treating PD Center.

### Late PD Stage

PD patients in the late stage are highly dependent on caregivers for daily living activities, owing to treatment-resistant motor symptoms or non-motor symptoms; these patients usually have a score on the Schwab and England Scale of <50% during periods of adequate symptomatic treatment (8). When a PD patient fits into the category of late-stage PD, the responsible neurologist informs the patient's General Practitioner.

### Data Collection and Retention

After obtaining informed consent, the patient's clinical data are collected at each scheduled visit and entered in the network database. Collection, storage and use of identifiable data and biological material beyond standard medical practice is performed in compliance with national and international guidelines. Data security should be part of the network's data management policy that includes retention, storage and disposal of health information. It should also include management of electronic and physical aspects, with appropriate steps taken to protect against intentional and inadvertent loss or breach. Access to health records should be protected by robust password control and regular password changes.

The network steering committee proposes and the general assembly approves the minimal clinical dataset to be shared by all network centers. This encompasses a set of rating scales, information on treatment and on relevant laboratory tests (see Table 3). In order to harmonize collection of clinical data, training for specific rating scales is provided by the network during dedicated training sessions. The network centers share a platform containing electronic case report forms to be filled when assessing patients. Collection of additional clinical data (including biobanking, imaging, etc.) may be performed by network centers who cooperate on specific research protocols.

An annual quality control of the data-entry process should be performed.

### Funding and Sustainability

Funding for the network functioning should be provided by the regional health government. While clinical activities are currently supported by the national health system, the network organization and functioning needs dedicated organizational and infrastructural resources. The business plan has to consider organizational, financial, and community sustainability, with periodic review and updates. Additional expenses for funding network activities are expected to be counterbalanced by the savings generated.

### Quality Assessment and Governance

Network performance is reviewed periodically with measures related to network efficacy and efficiency and to patient satisfaction. The shared platform containing electronic case report forms should contain a dashboard with updated performance information that is automatically displayed as clinical data are entered.

The panel reached consensus on the following measures that can be used to assess network performance: (1) Yearly consultations to emergency departments for PD patients followed by the network; (2) Yearly emergency admissions to neurological wards for PD patients followed by the network; (3) Efficacy of device-aided therapies (motor improvement 1 year after device-aided treatment compared to pre-treatment condition); (4) Waiting time at PD Centers and PD Clinics (waiting days before consultation by a PD network center); (5) Patient satisfaction questionnaires (marks given by PD patients and caregivers).

Governance can be provided by a network Steering Committee and a network General Assembly. The Steering Committee, composed by all PD Centers, elects a President and a Secretary with a two-year term. The President represents the network toward the regional Health government. The General Assembly, composed by all PD Clinics, meets yearly to review measures of outcome and to approve changes in the organization or functioning of the network.

## Discussion

The development of a regional PD network is expected to improve the standards of care and to optimize resources at the regional level. This is of high relevance, considering that the financial burden of PD on the society is quite high (18), with specific costs expected to increase more than the average health costs (19). Italian regions have a direct responsibility for governance and allocation of resources, regulate and organize health services and define financing criteria for regional HCPs. We provide a consensus agreement on the general organization based on clinical operational criteria, applicable to all HCPs accredited by the NHS. This model can serve as a basis to define the operational algorithm of health professions other than neurologists involved in PD care. This model has been implemented based on a political legislative decision and requires field-testing particularly to test its efficiency and advantages over standard practice.

A recent review showed that clinical networks can improve the delivery of healthcare (20). Coordinated and responsive care, tailored to the individual, with regular and timely medication reviews and information provision, is expected to improve the quality of life of PD patient. This is supported by the observation that patients who seek skilled care are at a lower risk of complications and have better quality of life (21), and that clinical networks can improve the delivery of healthcare (20). The hub-and-spoke organization of a PD network may increase the number of patients who receive early diagnosis and appropriate care. Predefined outcome measures contribute to the overall network quality.

A review of HCP facilities in Lombardy showed that PD Centers are mainly concentrated in and around Milan, with the northern and southeastern districts notably devoid of PD Centers. The first is a mountainous Alpine district; the latter is flat area bordering the neighboring Emilia region. More lenient criteria for PD Centers would only mildly mitigate such clustering around main towns (Figure 3). In the case of PD, where time-sensitive emergencies are uncommon, an uneven geographical representation of network centers may still be acceptable. In addition, telemedicine consultations may be performed by distant network centers and integrated within the PD network (22).

Few Italian regions have recently approved the design of regional PD networks. Apulia defined a regional networks that have some features in common with this consensus (23). The Piedmont region, instead, appointed two regional centers with expertise on DBS as network hubs, without delineating a detailed network structure (24). In both cases no dedicated resources were allocated for network activity, and quality measures of network performance were not defined. Other Italian regions have not yet deliberated on the structure of regional networks; some regions have consulted expert panels, and all are expected to proceed soon in accordance with a national measure on chronic diseases (1). Disease-centered networks provide an innovative opportunity to improve patient management, facilitate research and education on chronic neurological disease. We report a scientific consensus on the organization and implementation of a PD network in Lombardy that may serve as a first comprehensive organizational model. We provide a consensus definition of tertiary and secondary PD services and detail their interaction with the primary neurologist and the General Practitioner. The network structure depicted here may also apply to other chronic neurological conditions, such as dementias and amyotrophic lateral sclerosis. Regional disease networks may further cooperate at a national level, as foreseen by the national plan on chronic diseases (1). Agreement on a common structure may facilitate such cooperation.

A possible fallout of this consensus is the support of a sustainable healthcare systems. A structured network may reduce costs, improve timely access to treatment, facilitate earlier diagnosis, enhance patient outcomes, decrease hospital stays, and increase quality and duration of life. The network structure proposed here differs from other networks primarily aimed at sharing clinical experiences among professionals, such as the UK Parkinson's Excellence Network. This patient-funded initiative is mainly devoted to standardizing practices and sharing information. The Dutch ParkinsonNet was originally designed to train physical therapists who treat PD (25). Other research networks have different structures: the NS-Park lists 24 expert French centers designated by the Ministry of Health, the Kompetenznetz Parkinson is a German network of 40 movement disorder expert centers who conduct clinical trials on PD.

The availability of adequate resources is essential for network functioning. A solid infrastructure for data sharing must be created. The Lombardy region has an innovation technology company that can support the development of IT structure needed for the PD network. Support is also required for administration, training, and meetings. The initial investment is expected to be repaid by later savings on health resources, particularly after few years of operation.

## Data Availability Statement

Publicly available datasets were analyzed in this study. The list of Lombard HCPs can be found here: http://www.regione.lombardia.it/wps/portal/istituzionale/HCP/DettaglioServizio/servizi-e-informazioni/Cittadini/salute-e-prevenzione/strutture-sanitarie-e-sociosanitarie/ser-strutture-sanitarie-accreditate-sal/strutture-sanitarie-accreditate. Official statistical data on PD in Lombardy can be found here: https://www.dati.lombardia.it/stories/s/etgr-wnvm.

## Author Contributions

AA: conception, design, first draft, review. AD, VF, AF, MG, GM, CP, AP, GR, and MV: review of manuscript. DC: data search, data analysis, and review of manuscript. All authors contributed to the article and approved the submitted version.

## Conflict of Interest

The authors declare that the research was conducted in the absence of any commercial or financial relationships that could be construed as a potential conflict of interest. The handling editor declared a past co-authorship with the authors AF and AA.

## Abbreviations

ADL: Activity of daily living
HCP: Health-care provider
NHS: National health system
PD: Parkinson's disease.
